# Supplementary material for: Increased RBP4 and Asprosin Are Novel Contributors in Inflammation Process of Periodontitis in Obese Rats
Source: Int J Mol Sci. 2023 Nov 25;24(23):16739. doi: 10.3390/ijms242316739 (PMC10706687; doi:10.3390/ijms242316739)
Supplement: Supplementary file 1 [file ijms-24-16739-s001.zip › ijms-2694088-supplementary.pdf]

Table S1. Primer - sequence used in this experiment.

**Primer**	**Sequence (5′–3′)**
FBN1/asprosin	F 5′- CCTTCCGCTGTGTAAATACCT<br>R 5′- CTGCACACTCATCCTCATCTT
leptin	F 5′- CCTGTGGCTTTGGTCCTATC<br>R 5′- GATCCTGGTGACAATGGTCTT
Resistin/Retn	F 5′- CACTGTGTCCCATGGATGAA<br>R 5′- GGAGACTGACCAGCAATGTAG
adiponectin	F 5′- GCGCTCCTGTTCCTCTTAAT<br>R 5′- CATCCAACCTGCACAAGTTTC
Visfatin/nampt	F 5′- CTCCTTCAAGTGCAGCTATGT<br>R 5′- CCTGCTGGTGTCCTATGTAAAG
RBP4	F 5′- TGCAGACAGCTACTCCTTTG<br>R 5′- CCATTGTGCTCGATCCATCT
iNOS/Nos2	F 5′- CAACTACTGCTGGTGGTTACA<br>R 5′- AAGGTATGCCCGAGTTCTTTC
M-CSF	F 5′- CTTCATGCCAGATTGCCTTTG<br>R 5′- AAGCGCATGGTCTCATCTATT
IL-1**β**	F 5′- CAGTGAGGAGAATGACCTGTTC<br>R 5′- CGAGATGCTGCTGTGAGATT
TNF-**α**	F 5′- ACCACGCTCTTCTGTCTACT<br>R 5′- ATGATCTGAGTGTGAGGGTCT
IL-6	F 5′- GAAGTTAGAGTCACAGAAGGAGTG<br>R 5′- GTTTGCCGAGTAGACCTCATAG
CCL-2/MCP-1	F 5′- ATGAGTCGGCTGGAGAACTA<br>R 5′- ACTTCTGGACCCATTCCTTATTG
Col-1a1	F 5′- CAAGATGGTGGCCGTTACTAC<br>R 5′- GCTGCGGATGTTCTCAATCT
ALP	F 5′- ACAAGTGTGGCAGTGGTATT<br>R 5′- CTGCTTGAGGTTGAGGTTACA
Runx2	F 5′- TCATGGCCGGGAATGATGAG<br>R 5′- CGCTCCGGCCTACAAATCTC
RANKL	F 5′- CATCGCTCTGTTCCTGTACTT<br>R 5′- CGAGTCCTGCAAACCTGTAT
Pthlh	F 5′- CAACAAGGTGGAGACGTACAA<br>R 5′- TTCTCCTGTTCTCTGCGTTTC
Ctsk	F 5′- GAGGGCCAACTCAAGAAGAA<br>R 5′- TTGGAAGGCAGTGGTCATATAG
IL-17A	F 5′- TCAGCGTGTCCAAACACTGAG<br>R 5′- CGCCAAGCGAGTTAAAGACTT

β-actin	F 5′- GAGAGGGAAATCGTGCGTGAC<br>R 5′- CATCTGCTGGAAGGTGGACA
